# Postexercise cooling impairs muscle protein synthesis rates in recreational athletes

**DOI:** 10.1113/JP278996

**Published:** 2019-12-29

**Authors:** Cas J. Fuchs, Imre W. K. Kouw, Tyler A. Churchward‐Venne, Joey S. J. Smeets, Joan M. Senden, Wouter D. van Marken Lichtenbelt, Lex B. Verdijk, Luc J. C. van Loon

**Affiliations:** ^1^ Department of Human Biology, NUTRIM School of Nutrition and Translational Research in Metabolism Maastricht University Medical Centre+ Maastricht The Netherlands; ^2^ Department of Nutrition and Movement Sciences, NUTRIM School of Nutrition and Translational Research in Metabolism Maastricht University Medical Centre+ Maastricht The Netherlands

**Keywords:** cold‐water immersion, hydrotherapy, muscle protein synthesis, adaptation, stable isotope tracers, heavy water, deuterium oxide, resistance exercise

## Abstract

**Key points:**

- Protein ingestion and cooling are strategies employed by athletes to improve postexercise recovery and, as such, to facilitate muscle conditioning. However, whether cooling affects postprandial protein handling and subsequent muscle protein synthesis rates during recovery from exercise has not been assessed.
- We investigated the effect of postexercise cooling on the incorporation of dietary protein‐derived amino acids into muscle protein and acute postprandial (hourly) as well as prolonged (daily) myofibrillar protein synthesis rates during recovery from resistance‐type exercise over 2 weeks.
- Cold‐water immersion during recovery from resistance‐type exercise lowers the capacity of the muscle to take up and/or direct dietary protein‐derived amino acids towards *de novo* myofibrillar protein accretion. In addition, cold‐water immersion during recovery from resistance‐type exercise lowers myofibrillar protein synthesis rates during prolonged resistance‐type exercise training.
- Individuals aiming to improve skeletal muscle conditioning should reconsider applying cooling as a part of their postexercise recovery strategy.

**Abstract:**

We measured the impact of postexercise cooling on acute postprandial (hourly) as well as prolonged (daily) myofibrillar protein synthesis rates during adaptation to resistance‐type exercise over 2 weeks. Twelve healthy males (aged 21 ± 2 years) performed a single resistance‐type exercise session followed by water immersion of both legs for 20 min. One leg was immersed in cold water (8°C: CWI), whereas the other leg was immersed in thermoneutral water (30°C: CON). After water immersion, a beverage was ingested containing 20 g of intrinsically (l‐[1‐^13^C]‐phenylalanine and l‐[1‐^13^C]‐leucine) labelled milk protein with 45 g of carbohydrates. In addition, primed continuous l‐[*ring*‐^2^H_5_]‐phenylalanine and l‐[1‐^13^C]‐leucine infusions were applied, with frequent collection of blood and muscle samples to assess myofibrillar protein synthesis rates *in vivo* over a 5 h recovery period. In addition, deuterated water (^2^H_2_O) was applied with the collection of saliva, blood and muscle biopsies over 2 weeks to assess the effects of postexercise cooling with protein intake on myofibrillar protein synthesis rates during more prolonged resistance‐type exercise training (thereby reflecting short‐term training adaptation). Incorporation of dietary protein‐derived l‐[1‐^13^C]‐phenylalanine into myofibrillar protein was significantly lower in CWI compared to CON (0.016 ± 0.006 *vs*. 0.021 ± 0.007 MPE; *P = *0.016). Postexercise myofibrillar protein synthesis rates were lower in CWI compared to CON based upon l‐[1‐^13^C]‐leucine (0.058 ± 0.011 *vs*. 0.072 ± 0.017% h^−1^, respectively; *P = *0.024) and l‐[*ring*‐^2^H_5_]‐phenylalanine (0.042 ± 0.009 *vs*. 0.053 ± 0.013% h^−1^, respectively; *P = *0.025). Daily myofibrillar protein synthesis rates assessed over 2 weeks were significantly lower in CWI compared to CON (1.48 ± 0.17 *vs*. 1.67 ± 0.36% day^−1^, respectively; *P = *0.042). Cold‐water immersion during recovery from resistance‐type exercise reduces myofibrillar protein synthesis rates and, as such, probably impairs muscle conditioning.

## Key points

- Protein ingestion and cooling are strategies employed by athletes to improve postexercise recovery and, as such, to facilitate muscle conditioning. However, whether cooling affects postprandial protein handling and subsequent muscle protein synthesis rates during recovery from exercise has not been assessed.
- We investigated the effect of postexercise cooling on the incorporation of dietary protein‐derived amino acids into muscle protein and acute postprandial (hourly) as well as prolonged (daily) myofibrillar protein synthesis rates during recovery from resistance‐type exercise over 2 weeks.
- Cold‐water immersion during recovery from resistance‐type exercise lowers the capacity of the muscle to take up and/or direct dietary protein‐derived amino acids towards *de novo* myofibrillar protein accretion. In addition, cold‐water immersion during recovery from resistance‐type exercise lowers myofibrillar protein synthesis rates during prolonged resistance‐type exercise training.
- Individuals aiming to improve skeletal muscle conditioning should reconsider applying cooling as a part of their postexercise recovery strategy.

## Introduction

Resistance‐type exercise training is an effective strategy for increasing skeletal muscle mass and strength. A single session of resistance‐type exercise stimulates both muscle protein synthesis and breakdown rates, albeit the latter to a lesser extent (∼2–4‐fold lower increase). Accordingly, resistance‐type exercise improves net muscle protein balance, yet the balance remains negative in the absence of protein ingestion (Biolo *et al*. 1995; Phillips *et al*. 1997). Protein ingestion after exercise augments the increase in muscle protein synthesis rates and inhibits muscle protein breakdown rates, resulting in a positive net muscle protein balance during the acute stages of postexercise recovery (Biolo *et al*. 1997; Borsheim *et al*. 2002). Therefore, postexercise protein ingestion is widely applied by athletes as a strategy to increase postexercise muscle protein synthesis rates and, as such, to facilitate skeletal muscle conditioning. Current guidelines for protein intake after lower body resistance‐type exercise recommend the ingestion of 20 g (or 0.3 g kg^–1^ body mass) of a high quality protein source after exercise in healthy, young males (Moore *et al*. 2009; Witard *et al*. 2014; Moore, 2019).

Another strategy frequently applied by athletes to support postexercise skeletal muscle conditioning is cold‐water immersion (CWI). CWI, primarily as a result of its ability to decrease tissue temperature and blood flow (Gregson *et al*. 2011; Costello *et al*. 2012; Gregson *et al*. 2013; Mawhinney *et al*. 2013; Mawhinney *et al*. 2017a; Mawhinney *et al*. 2017b), has been suggested to reduce delayed onset muscle soreness (Leeder *et al*. 2012; Roberts *et al*. 2014; Hohenauer *et al*. 2015) and muscle oedema/swelling (Yanagisawa *et al*. 2003; Yanagisawa *et al*. 2004; Vaile *et al*. 2008; Roberts *et al*. 2014), improve recovery of muscle function/performance (Vaile *et al*. 2008; Vaile *et al*. 2011; Versey *et al*. 2013; Roberts *et al*. 2014) and increase gene expression of molecular markers of endurance exercise adaptation (Ihsan *et al*. 2014; Joo *et al*. 2016; Allan *et al*. 2017). However, it is important to note that not all studies show beneficial effects of CWI on markers of postexercise recovery (Paddon‐Jones & Quigley, 1997; Goodall & Howatson, 2008; Versey *et al*. 2013; Wilson *et al*. 2019). A recent meta‐analysis reported that the proposed beneficial effects of CWI on postexercise recovery seem to be based more upon subjective rather than objective (blood) markers (Hohenauer *et al*. 2015). Therefore, beneficial effects of CWI on postexercise recovery appear to be context specific and several factors, such as body composition, sex and training status, should also be taken into account (Myrer *et al*. 2001; Hohenauer *et al*. 2015; Stephens *et al*. 2016; Stephens *et al*. 2018).

So far, few studies have investigated the interaction between postexercise protein intake and cooling. Previously, Roberts *et al*. (2015) observed that combining postexercise cooling (CWI at ∼10°C for 10 min) with protein intake lowers anabolic signalling compared to combining active recovery (cycling at ∼37 W for 10 min) with protein intake in physically active, young men (Roberts *et al*. 2015). To date, no studies have assessed the effects of combining postexercise cooling with protein intake on the incorporation of dietary protein‐derived amino acids into skeletal muscle tissue and subsequent muscle protein synthesis rates. We hypothesized that postexercise cooling lowers postprandial muscle protein accretion and subsequent muscle protein synthesis rates during recovery in healthy, young men.

It could be speculated that potential negative effects of postexercise cooling on muscle protein synthesis are only transient and would not translate into a negative impact on muscle protein synthesis when assessed over a more prolonged period. Accordingly, we investigated the combined effects of more prolonged application of postexercise cooling and protein intake on muscle protein synthesis rates. In line with our first hypothesis, we hypothesized that postexercise cooling lowers myofibrillar protein synthesis rates during 2 weeks of resistance‐type exercise training in healthy, young men.

In the present study, we combined contemporary stable isotope tracer methodology with the ingestion of specifically produced intrinsically (l‐[1‐^13^C]‐phenylalanine and l‐[1‐^13^C]‐leucine) labelled milk protein to assess the acute effects of postexercise cooling on the incorporation of dietary protein‐derived amino acids into muscle protein and postprandial myofibrillar protein synthesis rates during 5 h of postexercise recovery. In addition, we applied oral deuterated water‐dosing methodology to determine the impact of combining postexercise cooling with protein intake on myofibrillar protein synthesis rates assessed over a 2 week exercise‐training period.

## Methods

### Ethical approval

This study was approved by the Medical Ethics Committee of the Maastricht University Medical Centre+ (METC 15‐3‐038) and conformed to the principles outlined in the *Declaration of Helsinki* for use of human subjects and tissue. Subjects were fully informed of the nature and possible risks of the experimental procedures before their written informed consent was obtained. This study was registered at clinicaltrials.gov as NCT02596542.

### Subjects

Twelve healthy young men (aged 21 ± 2 years) participated in the present study. All of the participants were considered recreationally active (exercising ∼3 times per week for a total duration of ∼4.5 h) and were familiar with resistance‐type exercise (but none were participating in structured resistance‐type exercise training). Subject characteristics are presented in Table 1. Participants had no prior history of participating in stable isotope amino acid tracer experiments and were deemed healthy based on their responses to a medical questionnaire.

**Table 1:** Subject characteristics

	Subjects (*n = *12)
Age (years)	21 ± 2
Body mass (kg)	76.2 ± 10.4
Height (cm)	184 ± 10
BMI (kg m^–2^)	22.6 ± 1.9
LBM (kg)	61.9 ± 7.3
CON leg lean mass (kg)	10.3 ± 1.5
CWI leg lean mass (kg)	10.2 ± 1.4
Whole body fat mass (kg)	12.7 ± 3.8
CON leg fat mass (kg)	2.5 ± 0.8
CWI leg fat mass (kg)	2.5 ± 0.7
Whole body fat mass (%)	16.1 ± 3.2
Leg press 1RM (kg)	274 ± 59
Leg extension 1RM (kg)	138 ± 29

Values are expressed as the mean ± SD. BMI, body mass index; LBM, lean body mass; 1RM, one repetition maximum. CWI, cold water immersion (8°C) leg. CON, thermoneutral water immersion (30°C) leg.

### General study design

**Figure 1:**
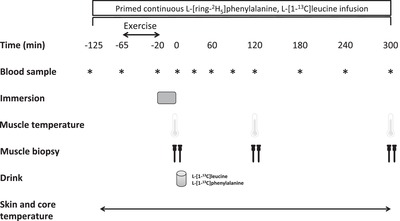
A schematic representation of the infusion day (day 0 of 2 week training programme; see Fig.  2) Participants performed leg press and leg extension exercise followed by water immersion of both legs (one leg was immersed in 8°C; the other leg was immersed in 30°C) for a total duration of 20 min. After muscle temperature measurements and collection of muscle biopsies from both legs, participants ingested 20 g of intrinsically labelled milk protein with 45 g of carbohydrates. Thereafter at *t = *120 and 300 min during postexercise recovery, muscle temperature measurements and muscle biopsies were collected from both legs. Blood samples, skin and core temperature measurements were collected throughout the infusion day.

**Figure 2:**
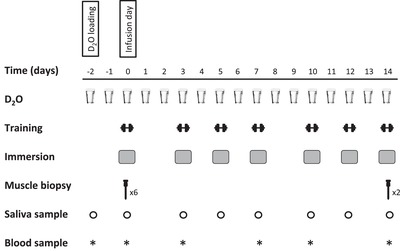
Experimental protocol for the 2 week training programme Two days before the start of the first training session (infusion day; see Fig. 1), participants performed the D_2_O loading day. Thereafter, D_2_O was provided every day during the 2 week training period. The 2 week training programme consisted of seven lower body resistance exercise sessions (leg press and extension machine). Every exercise session was followed by water immersion of both legs (one leg was immersed in 8°C; the other leg was immersed in 30°C) for a duration of 20 min. Bilateral muscle biopsies were collected after the first training session (infusion day; day 0) and after the last training session (day 14). Blood and saliva samples were collected throughout the 2 week training programme.

Each subject participated in one infusion day (Fig. 1) and a subsequent 2 week exercise‐training programme (Fig. 2). During the infusion day, the effect of postexercise cooling on postprandial muscle protein synthesis was studied following the ingestion of 20 g milk protein with 45 g of carbohydrates. At the start of the infusion day, primed continuous l‐[*ring*‐^2^H_5_]‐phenylalanine and l‐[1‐^13^C]‐leucine i.v. infusions were applied together with repeated blood sampling. After 1 h of rest, participants performed ∼45 min of resistance‐type exercise, after which they immersed both legs in water for 20 min (one leg at 8°C and one leg at 30°C). Thereafter, skeletal muscle biopsies from both legs were taken, before ingesting 20 g of intrinsically (l‐[1‐^13^C]‐phenylalanine and l‐[1‐^13^C]‐leucine) labelled milk protein with 45 g of glucose (polymers). In the subsequent 5 h recovery period, additional muscle biopsies were taken from the musculus vastus lateralis of both legs at 2 h and 5 h after drink ingestion (Fig. 1). During the 2 week exercise‐training programme, subjects performed progressive lower‐body resistance‐type exercise training (total of seven training sessions). Immediately after each exercise session, one leg was randomly assigned (the same leg as during the infusion day) to undergo 20 min of CWI (8°C: CWI), whereas the contralateral leg was immersed in thermoneutral water (30°C: CON). Deuterium‐oxide was ingested daily and muscle biopsy samples were obtained at the start (day 0) and at the end of the 2 week training period (day 14) to assess myofibrillar protein synthesis rates. More specifically, to calculate myofibrillar protein synthesis rates over the 2 week exercise‐training period, biopsies from both legs were used from the first training (infusion) day, 2 h after postexercise water immersion, and the final biopsies from both legs were taken after the last training session, again 2 h after postexercise water immersion.

### Pretesting

All subjects participated in a screening session, which was performed at least 1 week prior to the start of the experiment. First, each subject's body mass and height were measured, as well as body composition, by dual‐energy X‐ray absorptiometry (Discovery A; Hologic, Bedford, MA, USA). The system's software package (Hologic‐Apex software, version 4.5.3, with viewer software Hologic Physician's viewer, version 7.1) was used to determine whole body and regional lean and fat mass. Subsequently, thigh skinfold thickness was measured using Harpenden skinfold calipers (Baty International, Burgess Hill, UK) and divided by 2 to determine the thickness of the thigh s.c. fat layer over each participant's musculus vastus lateralis. In addition, participants were familiarized with the exercise equipment and performed maximum strength tests as determined by their one repetition maximum (1RM) for leg press and knee extension exercise using the multiple repetitions testing procedure (Mayhew *et al*. 1995) (Table 1). Subjects first performed a 5 min cycling exercise warm‐up at 100 W. Thereafter, for both leg press and extension, subjects performed two sets with 10 submaximal or warm‐up repetitions to become familiarized with the equipment and to have their lifting technique critiqued and corrected. Subjects then performed sets at progressively increasing loads until failing to complete a valid repetition, judged by their inability to complete the full range of motion for an exercise. A 2 min resting period between subsequent attempts was allowed. Finally, participants were familiarized with the water immersion procedure. One leg was immersed in cold water (8°C: CWI), whereas the other leg was immersed in thermoneutral water (30°C: CON) for a total duration of 20 min. Both legs were immersed to the level of the gluteal fold. The limb cooled (CWI) was randomized between each subject's dominant and nondominant leg and the same limb was cooled during the infusion day and after every exercise session during the 2 week training period. The contralateral leg was immersed in thermoneutral water after every exercise session. For the water immersion set‐up, two water tanks were used that were completely open at the top and contained a tap at the bottom. This allowed us to set and maintain water temperature (before the 20 min water immersion procedure) by adding water and/or ice from the top and remove water from below the tank. During water immersion, temperature was monitored and kept constant and still at 8°C in CWI and 30°C in CON. The same set‐up and procedures were applied for every water immersion session in the present study.

### Deuterated water‐dosing protocol (D_2_O loading)

Two days prior to initiation of the infusion day and thus the 2 week training period, subjects reported to the university for a deuterium oxide (D_2_O) dosing day. During this day, subjects ingested 8 × 50 mL boluses of 70% D_2_O (Cambridge Isotopes Laboratories, Andover, MA, USA) spaced evenly over the day. For the remainder of the 2 week experiment, subjects ingested 50 mL of deuterium oxide each day upon waking to maintain the enrichment. At the start of the dosing day, a basal saliva and blood sample was obtained together with saliva sample collection prior to all training sessions to monitor body water D_2_O enrichment during the 2 week exercise‐training programme.

### Diet and activity prior to the infusion trial and last exercise session

All subjects received the same standardized dinner (1710 kJ, consisting of 20.25 g of protein, 51.75 g of carbohydrate and 11.25 g of fat) the evening prior to the first (infusion day) and last day of the 2 week exercise‐training programme. All volunteers refrained from alcohol and any sort of additional exhaustive physical labor and/or exercise 2 days prior to the start (infusion day) and throughout the 2 week training programme. To assess compliance, subjects filled in food intake and physical activity questionnaires for 2 days prior to the start of the first (infusion day) and last day of the 2 week training programme.

### Experimental protocol for the infusion day (first day of the 2 week training programme)

The experimental protocol for the infusion day is outlined in Fig. 1. At the start of the infusion day at ∼07.30 h, following an overnight fast, subjects reported to the laboratory. First a telemetric pill (CorTemp HT150002; HQ Inc., Palmetto, FL, USA) was swallowed with tepid water for continuous measurements of body core temperature until the end of the experiment. In addition, ibuttons (Maxim Integrated Products, San Jose, CA, USA) were attached to the skin on the left and right upper thigh (∼10 cm above the patella) for continuous measurements of skin temperature during the entire trial. Thereafter, a polytetrafluoroethylene catheter was inserted into an antecubital vein for i.v. isotope tracer infusion and a second catheter was inserted in a dorsal hand vein of the contralateral arm, which was subsequently placed in a hot‐box (60°C) for ‘arterialized’ venous blood sampling. After baseline blood sample collection (*t* = –125 min), the plasma phenylalanine and leucine pools were primed with a single i.v. dose of L‐[*ring*‐^2^H_5_]‐phenylalanine (2.000 µmol kg^−1^) and l‐[1‐^13^C]‐leucine (3.991 µmol kg^−1^), respectively. Subsequently, an i.v. infusion of l‐[*ring*‐^2^H_5_]‐phenylalanine (infusion rate of 0.050 µmol kg^−1^ min^−1^) and l‐[1‐^13^C]‐leucine (0.100 µmol kg^−1^ min^−1^) was initiated and maintained until the end of the trial using a calibrated IVAC 598 pump (Cardinal Health, San Diego, CA, USA). After 1 h of supine rest, another arterialized blood sample (*t* = –65 min) was obtained. Subsequently, the participants performed a resistance‐type exercise session. After a 5 min warm‐up on a cycle ergometer at self selected intensity (∼129 W), the subjects performed four sets of 10 repetitions (at 80% 1RM) on both the leg press and knee extension exercise. After completion of the exercise bout (*t* = –20 min), another arterialized blood sample was obtained before the participants immersed both legs in water for a total duration of 20 min. Immediately after water immersion, another arterialized blood sample was obtained together with muscle temperature (MT23/5 probe; BAT‐10; Physitemp, Clifton, NJ, USA) measurements and muscle biopsies from both legs. The muscle temperature probe was inserted into the biopsy incision before each biopsy was collected from both legs. Immediately afterwards, the subjects ingested a recovery beverage (see below) at *t* = 0 min. Thereafter, repeated blood samples (*t* = 30, 60, 90, 120, 180, 240 and 300 min) were obtained together with muscle temperature measurements and biopsies from both legs at *t* = 120 min and *t* = 300 min. Muscle biopsies were collected from the middle region of the musculus vastus lateralis (∼15 cm above the patella) with a Bergström needle under local anaesthesia (Bergstrom, 1975). The first two biopsies in each leg (at *t* = 0 and 120 min) were taken from separate incisions. The difference between the separate incisions was ∼3 cm proximal from the previous incision. The last biopsy (*t* = 300 min) was collected from the same incision as the biopsy at *t* = 120 min. The biopsy at *t* = 300 min was collected with the needle inserted in a proximal direction. This method ensured that all biopsy sites were separated by at least 3 cm to minimize any artefact related to inflammation resulting from multiple biopsies. All biopsy samples were freed from any visible adipose tissue and blood, immediately frozen in liquid nitrogen and then stored at –80°C until subsequent analysis.

### Beverage

Subjects received a total beverage volume of 400 mL. The beverage contained 20 g of intrinsically (l‐[1‐^13^C]‐phenylalanine and l‐[1‐^13^C]‐leucine) labelled milk protein with 45 g of a vanilla flavored proprietary carbohydrate blend consisting of dextrose and maltodextrin (PepsiCo, Purchase, NY, USA). This was mixed in a bottle up to a total volume of 400 mL with water.

### Preparation of i.v. tracers

The stable isotope tracers l‐[*ring‐* ^2^H_5_]‐phenylalanine and l‐[1‐^13^C]‐leucine were purchased from Cambridge Isotopes (Andover, MA, USA) and dissolved in 0.9% saline before infusion (A15 Pharmacy, Gorinchem, The Netherlands).

### Experimental protocol for the 2 week training programme

The experimental protocol for the 2 week training programme is outlined in Fig. 2. Seven supervised training sessions (bilateral exercise) were performed on non‐consecutive days during 2 weeks. During every exercise day, first a saliva sample was collected (followed by blood sample collection at days 0, 3, 7, 10 and 14). After collection of body fluids, the exercise session started with a 5 min warm‐up on a cycle ergometer. After the warm‐up, the training session consisted of four sets of eight to 10 repetitions on both leg press and knee extension machines (Technogym, Rotterdam, The Netherlands). Initially, the workload was set at 80% of 1RM. When all 10 repetitions could be performed in four sets during an exercise session, workload was increased for the subsequent exercise session. Within 5 min of completing every exercise session, the recovery protocol (20 min water immersion in either 8°C (CWI) or 30°C (CON) water) was initiated. Immediately after water immersion, subjects ingested a drink containing 20 g of milk protein (MPC80; FrieslandCampina, Amersfoort, The Netherlands) and 45 g of a vanilla flavored proprietary carbohydrate blend consisting of dextrose and maltodextrin (PepsiCo). Subjects then remained in the laboratory and rested in the supine position for 2 h after completion of the recovery protocol to prevent reheating from showering or physical activity. For the last exercise day (day 14), subjects reported to the laboratory again at ∼07.30 h, following an overnight fast. First, basal saliva and blood samples were collected before the exercise session started followed by water immersion and drink ingestion. Two hours after drink ingestion, bilateral percutaneous needle muscle biopsy samples were taken from the musculus vastus lateralis, ∼15 cm above the patella. Both muscle biopsy samples were freed from any visible adipose tissue and blood, and immediately frozen in liquid nitrogen. Muscle samples were subsequently stored at –80°C until further analyses. All experimental sessions were conducted within the same temperature controlled laboratory (temperature: 21.1 ± 0.7°C and humidity: 41.9 ± 2.3%).

### Plasma and saliva analysis

Blood samples (10 mL) were collected in EDTA containing tubes and centrifuged at 1000 *g* and 4°C for 10 min. Aliquots of plasma were frozen in liquid nitrogen and stored at –80°C until analysis. Plasma glucose and insulin concentrations were analysed using commercially available kits (ref. no. A11A01667, Glucose HK CP; ABX Diagnostics, Montpellier, France; and ref. no. HI‐14K; Millipore, Billerica, MA, USA, respectively). Plasma amino acid concentrations and enrichments were determined by gas chromatography‐mass spectrometry analysis (GC‐MS) (Agilent 7890A GC/5975C; MSD, Wilmington, DE, USA). Specifically, internal standards of [U‐^13^C_6_]‐leucine, [U‐^13^C_9_^15^N]‐phenylalanine and [U‐^13^C_9_^15^N]‐tyrosine were added to the plasma samples. Plasma samples were deproteinized with dry 5‐sulphosalicylic acid. Free amino acids were purified using cation exchange chromatography (AG 50W‐X8 resin, mesh size: 100–200 µm, ionic form: hydrogen; Bio‐Rad Laboratories, Hercules, CA, USA). The purified amino acids were converted into *tert*‐butyldimethylsilyl (*tert*‐BDMS) derivatives with *N*‐tert‐butyldimethylsilyl‐*N*‐methyltrifluoroacetamide (MTBSTFA) before analysis by GC‐MS. The amino acid concentrations were determined using selective ion monitoring at mass/charge (*m/z*) 302 and 308 for unlabelled and [U‐^13^C_6_] labelled‐leucine, 336 and 346 for unlabelled and [U‐^13^C_9_^15^N] labelled phenylalanine respectively. The plasma leucine and phenylalanine ^13^C and ^2^H enrichments were determined at *m*/*z* 302 and 303 for unlabelled and labelled (1‐^13^C) leucine, respectively, and at *m*/*z* 336, 337 and 341 for unlabelled and labelled (1‐^13^C and *ring‐* ^2^H_5_) phenylalanine, respectively. The plasma free alanine mass isotopomers (M and M + 1) were determined using selective ion monitoring at *m*/*z* 232 and 233. Standard regression curves were applied from a series of known standard enrichment values against the measured values to assess the linearity of the mass spectrometer and to account for any isotope fractionation.

Body water enrichment was analysed using the saliva samples collected throughout the 2 week training programme. Saliva samples were collected at least 30 min after meal and drink ingestion. To collect saliva, subjects lightly chewed on a cotton swab (Celluron, Hartmann, Germany) for sufficient time to saturate the cotton swab with saliva. The swab was then removed and depressed using a syringe to extract the saliva into a sample tube. After collection, saliva was frozen in liquid nitrogen and stored at –80°C. All samples were centrifuged at 10 000 *g* to remove any debris. Following centrifugation, all samples were diluted 70‐fold with ddH_2_O to achieve deuterium enrichments within the detection limits of the isotope ratio mass spectrometer (IRMS). After dilution, samples were prepared for analysis on IRMS using the protocol reported by Scrimgeour *et al*. (1993) but with modifications. Briefly, a platinum rod (1091831; Thermo Fisher Scientific, Bremen, Germany) was placed inside each of the 12 mL glass vials (Labco Exetainer; Labco, Lampeter, UK). Three‐hundred microlitres of diluted saliva sample were then transferred into the vials. The glass vials were sealed using rubber septums and a screw cap. Air in each vial was simultaneously evacuated and replaced by 2% H_2_ in helium gas. The prepared vials were left at 21°C for a minimum of 48 h for deuterium equilibration to occur between the hydrogen gas and the saliva samples. The deuterium enrichment of the hydrogen gas was then measured in duplicate on a IRMS (Delta V Advantage with a Gasbench II; Thermo Fisher Scientific). Standard regression curves were applied from a series of known standard enrichment values against the measured values to assess the linearity of the mass spectrometer.

### Muscle analysis

Myofibrillar protein enriched fractions were extracted from ∼60 mg of wet muscle tissue by hand‐homogenizing on ice using a pestle in a standard extraction buffer (7 µL mg^−1^). The samples were spun at 700 *g* and 4°C for 15 min. The pellet was washed with 500 µL ddH_2_O and centrifuged at 700 *g* and 4°C for 10 min. The myofibrillar protein was solubilized by adding 1 mL of 0.3 m NaOH and heating at 50°C for 30 min with vortex mixing every 10 min. Samples were centrifuged at 9500 *g* and 4°C for 5 min and the supernatant containing the myofibrillar proteins was collected. The remaining pellet was washed with 1 mL of 0.3 m NaOH and then centrifuged at 9500 *g* and 4°C for 5 min. Subsequently, the supernatant was collected and added to the first supernatant. The remaining collagen pellet was discarded. Myofibrillar proteins were precipitated by the addition of 1 mL of 1 m perchloric acid and spinning at 700 *g* and 4°C for 10 min. The myofibrillar protein was washed twice with 70% ethanol and hydrolysed overnight in 2 mL of 6 m HCL at 110˚C. The free amino acids from the hydrolysed myofibrillar protein pellet were dried under a nitrogen stream at the same time as being heated to 110 °C. The free amino acids were then dissolved in 25% acetic acid solution, passed over cation exchange AG 50W‐X8 resin columns (mesh size: 100–200, ionic form: hydrogen; Bio‐Rad Laboratories) and eluted with 2 m NH_4_OH. The purified amino acids (l‐[1‐^13^C]‐phenylalanine and l‐[1‐^13^C]‐leucine enrichments) were analysed by GC‐C‐IRMS analysis. To determine myofibrillar protein l‐[1‐^13^C]‐phenylalanine and l‐[1‐^13^C]‐leucine enrichments by GC‐C‐IRMS analysis, the purified amino acids were converted into *N*‐ethoxycarbonyl ethyl ester derivatives with ethyl chloroformate. The derivatives were then measured by GC‐C‐IRMS (MAT 253; Finnigan, Bremen, Germany) using a DB5‐MS‐column (no. 122–5532; Agilent J+W Scientific GC Column, GC Isolink; Agilent Technologies, Santa Clara, CA, USA) and monitoring of ion masses 44, 45 and 46. For measurement of l‐[*ring*‐^2^H_5_]‐phenylalanine and [^2^H]alanine enrichment in the myofibrillar protein pools, the eluate was dried and the purified amino acids were derivatized to their *N*(*O*,*S*)‐ethoxycarbonyl ethyl esters. The derivatized samples were measured using a gas chromatography‐isotope ratio mass spectrometer (MAT 253; Thermo Fisher Scientific) equipped with a pyrolysis oven (GC‐P‐IRMS) using a 60 m DB‐17MS column and a 5 m precolumn (No. 122–4762; Agilent) and GC‐Isolink. Ion masses 1 and 2 were monitored to determine the ^2^H/^1^H ratios of muscle protein bound alanine. Standard regression curves were applied to assess the linearity of the mass spectrometer and to account for isotopic fractionation.

### Western blotting

Western blot analysis was performed on muscle tissue (*t* = 0, 2, and 5 h) from the infusion day (Fig. 1). A portion of each muscle sample frozen for biochemical analyses was homogenized in seven volumes Tris buffer (20 mm Tris‐HCL, 5 mm EDTA. 10 mm Na‐pyrosphospate, 100 mm NaF, 2 mm Na_3_VO_4_, 1% Nonident P‐40; pH 7.4) supplemented with protease and phosphatase inhibitors: aprotinin 10 µg mL^–1^, leupeptin 10 µg mL^–1^, benzamidin 3 mm and phenylmethylsulphonyl fluoride 1 mm. After homogenization, each muscle extract was centrifuged for 10 min at 10 000 *g* (4 °C) and sample buffer was added to the supernatant to final concentrations of 60 mm Tris, 10% glycerol, 20 mg mL^–1^ SDS, 0.1 mm dithiothreitol, 20 µg mL^–1^ bromophenol blue. The supernatant was then heated for 5 min at 100°C and immediately placed on ice. Immediately before analyses, the muscle extraction sample was warmed to 50 °C and centrifuged for 1 min at 1000 *g* [room temperature (RT)]. The total amount of sample loaded on the gel was based on protein content. After a Bradford assay, 30 µg protein were loaded in each lane. With the exception of mTOR, protein samples were run on a Criterion Precast TGX 4–20% gel (Order No. 567–1094; Bio‐Rad) ± 90 min at 150 V (constant voltage) and transferred onto a Trans‐blot Turbo 0.2 µm nitrocellulose membrane (Order No. 170–4159; Bio‐Rad) in 7 min at 2.5 A and 25 V. mTOR proteins were run and blotted for 10 min at 2.5 A and 25 V but on a Criterion Precast XT 3–8% Tris‐acetate gel (Order No. 345‐0130; Bio‐Rad). Specific proteins were detected by overnight incubation at 4°C on a shaker with specific antibodies in 50% in PBS Odyssey blocking buffer (Part No. 927‐40 000; Li‐Cor Biosciences, Lincoln, NE, USA) after blocking for 60 min at RT in 50% in PBS Odyssey blocking buffer. Polyclonal primary phospho‐specific antibodies, anti‐phospho‐mTOR (Ser^2448^), anti‐phospho‐S6K1 (Thr^389^), anti‐phospho‐S6K1 (Thr^421^/Ser^424^), anti‐phospho‐rpS6 (Ser^240^/Ser^244^), anti‐phospho‐rpS6 (Ser^235^/Ser^236^) and anti‐phospho‐4E‐BP1 (Thr^37/46^) were purchased from Cell Signaling Technology (Danvers, MA, USA). In addition, anti‐mTOR, anti‐S6K1, anti‐RS6 and anti‐4E‐BP1 were also purchased from Cell Signaling Technology. Following incubation, membranes were washed three times 10 min in 0.1% PBS‐Tween 20 and once for 10 min in PBS. Next, samples were incubated on a shaker (1 h at RT) with infrared secondary antibodies, donkey anti‐rabbit IRDYE 800 (dilution 1:10 000; Cat. No. 611‐732‐127; Rockland Immunochemicals, Pottstown, PA, USA) and donkey anti‐mouse IRDYE 800CW (dilution 1:10 000; Cat. No. 626–32 212; Li‐Cor Biosciences) dissolved in 50% PBS Odyssey blocking buffer. After a final wash step (3 × 10 min) in 0.1% Tween 20‐PBS and once 10 min in PBS, protein quantification was performed by scanning on an Odyssey Infrared Imaging System (Li‐Cor Biosciences). Ponceau S staining was used to standardize for the amount of protein loaded. Phosphorylation status as a proxy of activation of the signalling proteins was expressed relative to the total amount of each protein.

### mRNA analyses

mRNA analysis was performed on muscle tissue (*t* = 0, 2 and 5 h) from the infusion day (Fig. 1). Total RNA was isolated from 10–20 mg of frozen muscle tissue using TRIzol^®^ Reagent (Life Technologies, Invitrogen, Carlsbad, CA, USA), in accordance with the manufacturer's instructions. Total RNA quantification was carried out spectrophotometrically at 260 nm (NanoDrop ND‐1000 Spectrophotometer; Thermo Fisher Scientific) and RNA purity was determined as the ratio of readings at 260/280 nm. Thereafter, first strand cDNA was synthesized from 1 µg of RNA sample using iScript™ cDNA synthesis kit (Bio‐Rad; Cat. No. 170–8891). Taqman PCR was carried out using a 7300 Real Time PCR System (Applied Biosystems, Foster City, CA, USA), with 2 µL of cDNA, 12.5 µL of Taqman™ master mix, 1.25 µL of Taqman™ probe and 9.25 µL of H_2_O in a 25 µL final well volume. Each sample was run in duplicate, together with a serial dilution standard curve. The housekeeping gene 18S was used as an internal control. Taqman primer/probe sets were obtained from Applied Biosystems: FOXO1 (Hs 0 105 4576_m1), MuRF1 (Hs 002 61590_m1), MAFbx (Hs 0 104 1408_m1), mTOR (Hs 002 34508_m1), p70S6K (Hs 001 77357_m1), GLUT4 (Hs 001 68966_m1), TNF‐α (Hs 0 111 3624_g1), IL‐6 (Hs 009 85639_m1), LAT1/SLC (Hs 001 85826_m1), PAT1 (Hs 0 109 2773_m1), SNAT2 (Hs 0 108 9954_m1), CD98 (Hs 003 74243_m1) and 18S (Hs 0 300 3631_g1). The thermal cycling conditions used were: 2 min at 50 °C, 10 min at 95 °C, followed by 40 cycles at 95 °C for 15 s and 60 °C for 1 min. Ct values of the target genes were normalized to Ct values of the internal control and final results were calculated as relative expression against the standard curve.

### Calculations

Ingestion of l‐[1‐^13^C]‐phenylalanine labelled protein, i.v. infusion of l‐[*ring*‐^2^H_5_]‐phenylalanine and blood sample enrichment values were used to calculate total and exogenous phenylalanine rates of appearance (*R*_a_), as well as plasma availability of dietary protein‐derived phenylalanine that appeared in the systemic circulation as a fraction of the total amount of phenylalanine ingested (Phe*_plasma_*). For these calculations, modified Steele's equations (in non‐steady state conditions) were used (Boirie *et al*. 1996; Dangin *et al*. 2003).

These parameters were calculated as:

(1) $$\mathrm{Total}\;R_{a}=\frac{F_{\mathrm{iv}}-\left[pV\cdot C\left(t\right)\cdot \frac{dE_{\mathrm{iv}}}{dt}\right]}{E_{iv}\left(t\right)}$$

(2) $$\mathrm{Exo}R_{a}=\frac{\mathrm{Total}\;R_{a}\cdot E_{\mathrm{po}}\left(t\right)+\left[pV\cdot C\left(t\right)\cdot \frac{dE_{\mathrm{po}}}{dt}\right]}{E_{\mathrm{prot}}}$$

(3) $$\mathrm{Ph}e_{\mathrm{plasma}}=\left(\frac{{\mathrm{AUC}}_{\mathrm{ExoRa}}}{{\mathrm{Phe}}_{\mathrm{prot}}}\right)\cdot 100$$

where *F*_iv_ is the intravenous tracer infusion rate (µmol kg^−1^ min^−1^), *pV* (0.125 L kg^−1^) is the distribution volume for phenylalanine (Boirie *et al*. 1996), *C(t)* is the mean plasma phenylalanine concentration between two consecutive time points, *dE*_iv_/*dt* represents the time‐dependent variation of plasma phenylalanine enrichment derived from the intravenous tracer and *E*_iv_ *(t)* is the mean plasma phenylalanine enrichment from the intravenous tracer between two consecutive time points. Exo*R*_a_ represents the plasma entry rate of dietary phenylalanine, *E*_po_ *(t)* is the mean plasma phenylalanine enrichment for the ingested tracer, *dE*_po_/*dt* represents the time‐dependent variations of plasma phenylalanine enrichment derived from the oral tracer and *E*_prot_ is the l‐[1‐^13^C]‐phenylalanine enrichment in the dietary protein. Phe_plasma_ is the percentage of ingested dietary phenylalanine that becomes available in the plasma and is calculated using *Phe*_Prot_ and *AUC*_ExoRa_. *Phe*_Prot_ is the amount of dietary phenylalanine ingested and *AUC*_ExoRa_ represents the area under the curve (AUC) of Exo*R*_a_, which corresponds to the amount of dietary phenylalanine that appeared in the blood over a 5 h period following ingestion.

The fractional synthesis rate (FSR) of myofibrillar protein was calculated by dividing the increment in enrichment in the product (i.e. protein‐bound l‐[1‐^13^C]‐leucine, l‐[*ring*‐^2^H_5_]‐phenylalanine or [^2^H]‐alanine) by the enrichment of the respective precursor enrichments (i.e. plasma free amino acids or mean body water deuterium enrichment corrected by a factor of 3.7) (Holwerda *et al*. 2018). Weighted mean plasma l‐[*ring*‐^2^H_5_]‐phenylalanine and l‐[1‐^13^C]‐leucine enrichments were used as the preferred precursor pools to estimate myofibrillar protein fractional synthesis rates from the continuously infused l‐[*ring*‐^2^H_5_]‐phenylalanine and l‐[1‐^13^C]‐leucine tracers. Consequently, myofibrillar *FSR* was calculated as:

(4) $$\mathrm{FSR}\left(\%\cdot h^{-1}or\;\%\cdot d^{-1}\right)=\left(\frac{E_{m2}-E_{m1}}{E_{\mathrm{precursor}}\times t}\right)\times 100$$

where *E*_m2_ *– E*_m1_ represents muscle protein‐bound l‐[*ring*‐^2^H_5_]‐phenylalanine, l‐[1‐^13^C]‐leucine or [^2^H]‐alanine. *E*_precursor_ represents the average plasma l‐[*ring*‐^2^H_5_]‐phenylalanine or l‐[1‐^13^C]‐leucine enrichment (for the infusion day) or the corrected mean body water deuterium enrichment (for the 2 week training programme) during the tracer incorporation period. *t* indicates the time interval (hours or days) between biopsies.

### Statistical analysis

Unless otherwise stated, all data are expressed as the mean ± SD. Changes in body water deuterium enrichments, blood glucose and insulin, plasma amino acid concentrations and enrichments, exogenous phenylalanine *R*_a_, and core body temperature were analysed using one‐way repeated‐measures ANOVA with time as within‐subjects factor. A two‐factor (treatment × time) repeated‐measures ANOVA was performed for the analysis of l‐[1‐^13^C]‐phenylalanine myofibrillar protein‐bound enrichments, skeletal muscle and skin temperature, anabolic signalling and gene expression. A Student's paired *t* test was performed for the analysis of FSR values. In case of a significant main effect of time or time × treatment interaction, Bonferroni corrected pairwise comparisons were performed where appropriate. Cohen's effect size (*d*) and 95% confidence intervals (CI) of the differences between CWI and CON were calculated for the primary outcomes variables. In addition, Cohen's effect sizes (*d*) were also calculated when significant differences between CWI and CON were observed for markers of anabolic signalling and gene expression. Effect sizes of 0.2 are considered small, 0.5 are considered medium, and 0.8 are considered large (Cohen, 1988). *P *< 0.05 was considered statistically significant. All calculations were performed using SPSS, version 24.0 (IBM Corp., Armonk, NY, USA).

## Results

### Thermoregulatory responses

**Figure 3:**
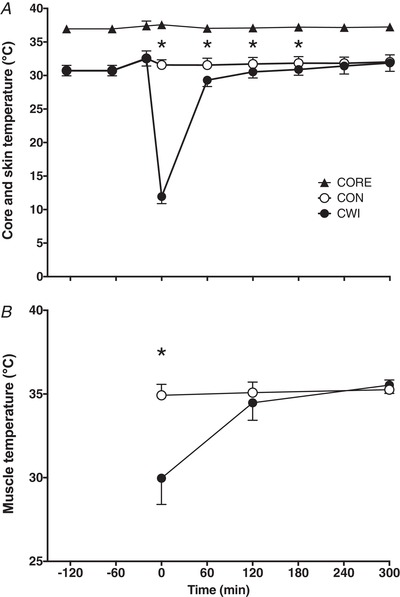
Core, skin and muscle temperature Core and skin temperature (*A*) during the entire experimental protocol and muscle temperature (*B*) immediately after water immersion (*t = *0) and *t = *120 and 300 min after drink ingestion in CON and CWI in healthy young men (*n = *12). Values represent the mean ± SD. ^*^Significantly different (*P *< 0.001) from CON. CWI, cold water immersion (8°C) leg; CON, thermoneutral water immersion (30°C) leg; CORE, core temperature.

Core and thigh skin temperature over the entire experiment are shown in Fig. 3*A*. Core temperature slightly increased after water immersion (time effect, *P *< 0.01) but did not change significantly throughout the remainder of the experiment. A significant time × treatment interaction was observed for thigh skin temperature (*P *< 0.001). Thigh skin temperature was significantly higher after exercise in both the CWI and CON leg (*P *< 0.001), after which it decreased back to baseline values following water immersion and did not change throughout the remainder of the experiment in the CON leg. For the CWI leg, thigh skin temperature was significantly reduced immediately after water immersion by ∼20.5°C (*P *< 0.001) after which it returned towards baseline values over the remainder of the experiment. Thigh skin temperature was significantly different between the CWI and CON legs immediately after water immersion up to 3 h after ingestion of the recovery beverage (*P *< 0.001).

Muscle temperature of both legs after water immersion is shown in Fig. 3*B*. Considering a ∼0.5 cm skinfold thickness, the muscle temperature probe (5 cm) was inserted in the muscle at a depth of ∼4.5 cm. A significant time × treatment interaction was observed for muscle temperature (*P *< 0.001). Muscle temperature did not change over time in the CON leg, although it was significantly different between 0 and 120, 120 and 300, as well as 0 and 300 min, in the CWI leg (*P *< 0.01). After water‐immersion (*t* = 0 min), muscle temperature in the CWI leg was significantly lower (∼5°C) compared to the CON leg (*P *< 0.001). At time points 120 min and 300 min, muscle temperature was no longer significantly different between legs.

### Plasma and saliva analysis

Plasma glucose concentrations significantly increased from *t* = 0 to *t* = 30 min (from ∼4.9 to ∼7.1 mmol L^–1^; time effect, *P *< 0.001). At other time points no significant differences were observed compared to baseline (*t* = 0 min) (data not shown). Plasma insulin concentrations were significantly increased from *t* = 0 (∼8.5 mU L^–1^) to *t* = 30 (∼68.4 mU L^–1^) and 60 min (∼24.9 mU L^–1^) (time effect, *P *< 0.05). At other time points after drink ingestion, no significant differences were observed compared to baseline (*t* = 0 min) (data not shown). Both plasma phenylalanine and leucine concentrations increased following drink ingestion (time effect, *P *< 0.001) and remained above basal levels for 120 min (data not shown).

During the postabsorptive period, plasma l‐[ring‐^2^H_5_]‐phenylalanine and l‐[1‐^13^C]‐leucine remained in a steady state at ∼6.5–7.0 and ∼5.0–5.5 MPE, respectively (data not shown). Following drink ingestion, plasma l‐[ring‐^2^H_5_]‐phenylalanine enrichments were significantly lower for 60 min before returning to fasting steady‐state levels (time effect, *P *< 0.001), whereas plasma l‐[1‐^13^C]‐leucine enrichments increased in response to drink ingestion (time effect, *P *< 0.001) and remained at an elevated steady state of ∼6.0–8.0 MPE for the duration of the postprandial period. Following drink ingestion, plasma l‐[1‐^13^C]‐phenylalanine enrichments increased rapidly (time effect, *P *< 0.001) from ∼0.0 to ∼13.0 MPE after 30 min and began to decline thereafter, remaining elevated above fasting levels for the remainder of the postprandial period (data not shown).

**Figure 4:**
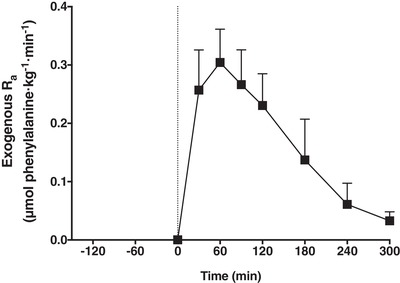
Exogenous phenylalanine rate of appearance Exogenous phenylalanine *R*_a_ in healthy young men (*n = *12). The dotted line represents the ingestion of 20 g of intrinsically labelled milk protein with 45 g of carbohydrate (at *t = *0 min). Values represent the mean ± SD.

Ingestion of the 20 g of intrinsically labelled milk protein resulted in a rapid rise in exogenous phenylalanine appearance rates (time effect, *P *< 0.001) (Fig. 4) and this remained significantly elevated compared to baseline (*t* = 0 min) throughout the 5 h postprandial period. Over the entire 5 h period, 14.1 ± 1.2 g (71 ± 6%) of the ingested protein derived amino acids had been released into the circulation.

Body water deuterium enrichment (data not shown) over the 2 week exercise‐training period slightly increased (time effect, *P *< 0.001) over the duration of the experiment and averaged 0.72 ± 0.10%.

### Muscle analysis

**Figure 5:**
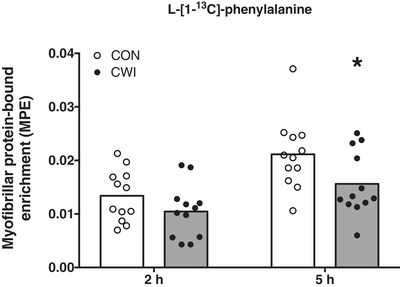
L‐[1‐^13^C]‐phenylalanine incorporation into myofibrillar protein after drink ingestion l‐[1‐^13^C]‐phenylalanine incorporation into myofibrillar protein after drink ingestion with intrinsically labelled l‐[1‐^13^C]‐phenylalanine in healthy young men (*n = *12). Bars are means and dots represent individual values. ^*^Significantly different (*P = *0.016) from CON. MPE, mole percent excess; CWI, cold water immersion (8°C) leg; CON, thermoneutral water immersion (30°C) leg.

Myofibrillar l‐[1‐^13^C]‐phenylalanine enrichments are shown in Fig. 5. A significant time × treatment interaction was observed for myofibrillar l‐[1‐^13^C]‐phenylalanine enrichments (*P = *0.021). Myofibrillar l‐[1‐^13^C]‐phenylalanine enrichments in both the CWI and CON leg were significantly increased from *t* = 0 to 2 h (0.011 ± 0.005 and 0.013 ± 0.005 MPE, respectively; *P *< 0.001) and 5 h (0.016 ± 0.006 and 0.021 ± 0.007 MPE, respectively; *P *< 0.001). Myofibrillar l‐[1‐^13^C]‐phenylalanine enrichments did not differ between the CWI and CON leg at 2 h (*P = *0.132; *d = *0.46; 95% CI = –0.007 to 0.001 MPE). However, myofibrillar l‐[1‐^13^C]‐phenylalanine enrichments were lower in the CWI leg compared to the CON leg at 5 h (*P = *0.016; *d = *0.83; 95% CI = –0.010 to –0.001 MPE).

**Figure 6:**
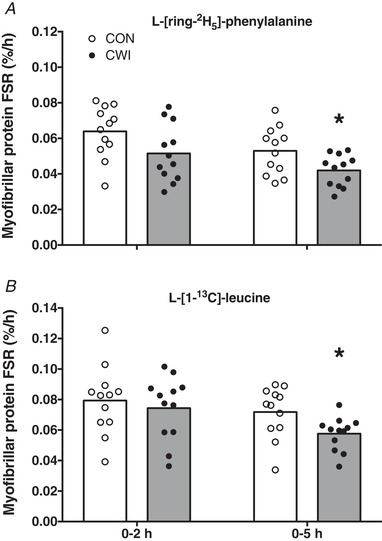
Myofibrillar protein synthesis rates during 5 h of postexercise recovery Myofibrillar protein FSRs as calculated with l‐[ring‐^2^H_5_]‐phenylalanine (*A*) or l‐[1‐^13^C]‐leucine (*B*) as tracer during 5 h of postexercise recovery with the ingestion of 20 g of intrinsically labelled milk protein with 45 g of carbohydrate in healthy young men (*n = *12). Bars are means and dots represent individual values. ^*^Significantly different (*P *< 0.05) from CON. FSR, fractional synthetic rate; CWI, cold water immersion (8°C) leg; CON, thermoneutral water immersion (30°C) leg.

**Figure 7:**
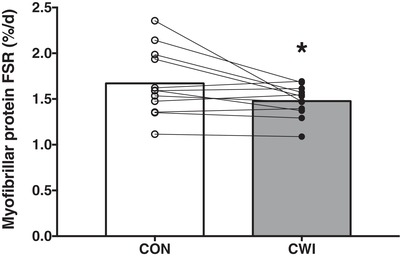
Myofibrillar protein synthesis rates during 2 weeks of resistance training Myofibrillar protein FSR as calculated using body water deuterium as precursor during 2 weeks of resistance training with or without CWI in healthy young men (*n = *12). Bars are means and dots represent individual values. ^*^Significantly different (*P = *0.042) from CON. FSR, fractional synthetic rate. CWI, cold water immersion (8°C) leg; CON, thermoneutral water immersion (30°C) leg.

Myofibrillar protein FSR values based on l‐[*ring*‐^2^H_5_]‐phenylalanine infusion with plasma l‐[*ring*‐^2^H_5_]‐phenylalanine enrichments as precursor (Fig. 6*A*) or using l‐[1‐^13^C]‐leucine infusion and ingestion with plasma l‐[1‐^13^C]‐leucine enrichments as precursor (Fig. 6*B*) are shown in Fig. 6. Myofibrillar protein FSR as calculated over 0–2 h did not differ between the CWI and CON leg based on the l‐[*ring*‐^2^H_5_]‐phenylalanine tracer (0.052 ± 0.016 *vs*. 0.064 ± 0.015% h^−1^, respectively; *P = *0.085; *d = *0.55; 95% CI = –0.027 to 0.002% h^−1^) and the l‐[1‐^13^C]‐leucine tracer (0.074 ± 0.021 *vs*. 0.079 ± 0.022% h^−1^, respectively; *P = *0.455; *d = *0.16; 95% CI = –0.019 to 0.009% h^−1^). Myofibrillar protein FSR as calculated over 0–5 h was significantly lower in the CWI compared to the CON leg based on the l‐[*ring*‐^2^H_5_]‐phenylalanine tracer (0.042 ± 0.009 *vs*. 0.053 ± 0.013% h^−1^, respectively; *P = *0.025; *d = *0.75; 95% CI = –0.020 to –0.002% h^−1^), as well as the l‐[1‐^13^C]‐leucine tracer (0.058 ± 0.011 *vs*. 0.072 ± 0.017% h^−1^, respectively; *P = *0.024; *d = *0.75; 95% CI = –0.026 to –0.002% h^−1^). Myofibrillar protein FSR (% day^–1^) over the 2 week training period was calculated using mean body water deuterium enrichment corrected by a factor of 3.7 as precursor and is shown in Fig. 7. In accordance with the acute myofibrillar protein FSR measurements, myofibrillar protein synthesis rates over 2 weeks were significantly lower in the CWI compared to the CON leg (1.48 ± 0.17 *vs*. 1.67 ± 0.36% day^−1^, respectively; *P = *0.042; *d = *0.67; 95% CI = –0.38 to –0.01% h^−1^).

### Anabolic signalling and gene expression

**Figure 8:**
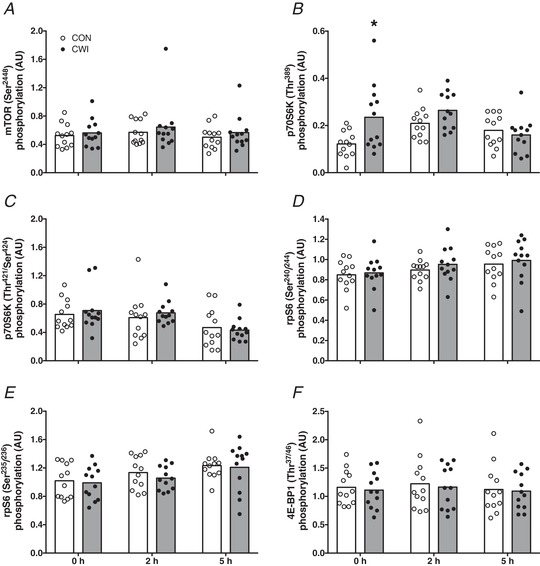
Anabolic signalling Skeletal muscle phosphorylation status (ratio of phosphorylated to total protein) of mTOR (Ser2448) (*A*), p70S6K (Thr389) (*B*), p70S6K (Thr421/Ser424) (*C*), rpS6 (Ser240/244) (*D*), rpS6 (Ser235/236) (*E*) and 4E‐BP1 (Thr37/46) (*F*) immediately after postexercise water immersion (*t = *0 h) and after ingestion of 20 g of intrinsically labelled milk protein with 45 g of carbohydrate (*t = *2 and 5 h) in healthy young men (*n = *12). Bars are means and dots represent individual values. ^*^Significantly different (*P *< 0.05) from CON. CWI, cold water immersion (8°C) leg; CON, thermoneutral water immersion (30°C) leg.

The phosphorylation status (ratio of phosphorylated to total protein) of key proteins involved in the initiation of muscle protein synthesis are shown in Fig. 8. No significant differences were observed for muscle mTOR (Ser2448) phosphorylation status (Fig. 8*A*). A significant time × treatment interaction was observed for muscle p70S6K (Thr389) phosphorylation status (*P *< 0.05) (Fig. 8*B*). Muscle p70S6K (Thr389) phosphorylation status was significantly decreased from 2 to 5 h (*P = *0.021) in the CWI leg and significantly increased from 0 to 2 h (*P = *0.001) in the CON leg, with a significantly higher phosphorylation status at *t* = 0 h in the CWI compared to the CON leg (*P = *0.016; *d = *0.86). Muscle p70S6K (Thr421/Ser424) phosphorylation status (Fig. 8*C*) was significantly decreased from 2 to 5 h (*P = *0.003) and from 0 to 5 h (*P = *0.006) in both the CWI and CON leg, with no significant treatment effect or time × treatment interaction observed. Muscle rpS6 (Ser240/244) phosphorylation status (Fig. 8*D*) was significantly increased at both the 2 h (*P = *0.004) and 5 h (*P = *0.033) time point compared to 0 h for both the CWI and CON leg, with no significant treatment effect or time × treatment interaction observed. Muscle rpS6 (Ser235/236) phosphorylation status (Fig. 8*E*) was significantly increased at both the 2 h (*P = *0.017) and 5 h (*P = *0.004) time point compared to 0 h for both the CWI and CON leg, with no significant treatment effect or time × treatment interaction observed. No significant differences were observed for muscle 4E‐BP1 (Thr37/46) phosphorylation status (Fig. 8*F*).

**Figure 9:**
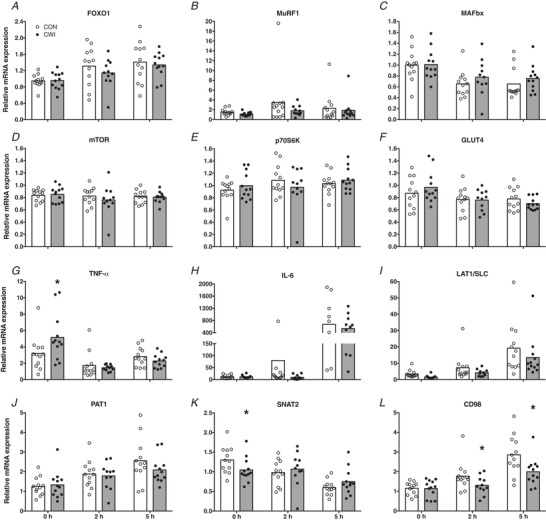
Gene expression Skeletal muscle mRNA expression of FOXO1 (*A*), MuRF1 (*B*), MAFbx (*C*), mTOR (*D*), p70S6K (*E*), GLUT4 (*F*), TNF‐α (*G*), IL‐6 (*H*), LAT1/SLC (*I*), PAT1 (*J*), SNAT2 (*K*) and CD98 (*L*) immediately after postexercise water immersion (*t = *0 h) and after ingestion of 20 g of intrinsically labelled milk protein with 45 g of carbohydrate (*t = *2 and 5 h) in healthy young men (*n = *12). Bars are means and dots represent individual values. ^*^Significantly different (*P *< 0.05) from CON. CWI, cold water immersion (8°C) leg; CON, thermoneutral water immersion (30°C) leg.

Skeletal muscle mRNA expression for selected genes implicated in the regulation of muscle mass, intracellular amino acid and glucose transport and inflammation are shown in Fig. 9. No treatment effects and time × treatment interactions were observed for muscle FOXO1 (Fig. 9*A*), MuRF1 (Fig. 9*B*), MAFbx (Fig. 9*C*), mTOR (Fig. 9*D*), P70S6K (Fig. 9*E*), GLUT4 (Fig. 9*F*), IL‐6 (Fig. 9*H*) and PAT1 (Fig. 9*J*) mRNA expression. Over time, muscle FOXO1 (2 h: *P = *0.034; 5 h: *P = *0.007) and PAT1 (2 h: *P = *0.010; 5 h: *P = *0.005) mRNA expression were significantly increased at both the 2 h and 5 h time point (Fig. 9*A* and 9*J*, respectively), whereas muscle MAFbx (2 h: *P = *0.003; 5 h: *P = *0.033) and GLUT4 (2 h: *P = *0.019; 5 h: *P = *0.037) mRNA expression (Fig. 9*C*–*F*, respectively) were significantly decreased at both the 2 h and 5 h time point compared to 0 h for both the CWI and CON leg. Muscle IL‐6 mRNA expression (Fig. 9*H*) was significantly higher at 5 h compared to 0 h (*P *< 0.001) and 2 h (*P *< 0.001) for both the CWI and CON leg. Muscle LAT1/SLC mRNA expression (Fig. 9*I*) was significantly higher at 5 h compared to 0 h (*P = *0.016), with a significant treatment effect (*P = *0.009) but no time × treatment interaction observed. A significant time × treatment interaction was observed for muscle TNF‐α (*P = *0.001) (Fig. 9*G*), SNAT2 (*P = *0.018) (Fig. 9*K*) and CD98 (*P = *0.015) (Fig. 9*L*) mRNA expression. Muscle TNF‐α mRNA expression was significantly decreased at time point 2 h (*P = *0.002) and 5 h (*P = *0.025) compared to 0 h and was significantly higher at 5 h compared to 2 h (*P = *0.019) in the CWI leg. In addition, muscle TNF‐α mRNA expression was significantly higher at 5 h compared to 2 h (*P = *0.033) in the CON leg. Muscle TNF‐α mRNA expression was significantly higher at *t* = 0 h in the CWI leg compared to the CON leg (*P = *0.004; *d = *1.04). Muscle SNAT2 mRNA expression was significantly lower at 5 h compared to 0 h in both the CWI (*P = *0.031) and the CON (*P *< 0.001) leg, and at 5 h compared to 2 h (*P = *0.002) in the CON leg only. Muscle SNAT2 mRNA expression was significantly lower at *t* = 0 h in the CWI leg compared to the CON leg (*P = *0.037; *d = *0.68). Muscle CD98 mRNA expression was significantly higher at 5 h compared to 0 h (*P = *0.024) and 2 h (*P = *0.010) in the CWI leg. For the CON leg, muscle CD98 mRNA expression was significantly higher at 2 h compared to 0 h (*P = *0.018) and at 5 h compared to both 0 h (*P = *0.001) and 2 h (*P = *0.011). Muscle CD98 mRNA expression was significantly lower at *t* = 2 (*P = *0.012; *d = *0.79) and 5 h (*P = *0.007; *d = *1.03) in the CWI leg compared to the CON leg.

## Discussion

In the present study, we assessed the impact of postexercise cooling on postprandial myofibrillar protein synthesis rates during recovery from resistance‐type exercise. Cooling lowered skin and muscle temperature and blunted the postexercise increase in myofibrillar protein synthesis rate, with less dietary protein‐derived amino acids being taken up and used for *de novo* myofibrillar protein synthesis. Furthermore, when applied over a 2 week period, postexercise cooling also resulted in significantly lower daily myofibrillar protein synthesis rates.

In the present study, we provided a bolus of 20 g of intrinsically labelled milk protein (together with 45 g of carbohydrates) after resistance‐type exercise. In line with previous research (Gorissen *et al*. 2014; Groen *et al*. 2015; Trommelen *et al*. 2016), we showed that the dietary protein‐derived amino acids are effectively being taken up and released in the systemic circulation (Fig. 4), thereby providing precursors for *de novo* myofibrillar protein synthesis (Fig. 5). In total, more than 70% of the ingested protein derived amino acids were released in the circulation, strongly increasing plasma amino acid concentrations.

In addition to dietary protein ingestion, postexercise cooling is a strategy applied by many athletes to support postexercise recovery, with CWI being a popular form of cooling (Ihsan *et al*. 2016; Broatch *et al*. 2018). However, there is now evidence to suggest that postexercise CWI blunts important molecular pathways involved in the regulation of myofibrillar protein synthesis, such as anabolic signalling and ribosomal biogenesis (Roberts *et al*. 2015; Figueiredo *et al*. 2016; Fyfe *et al*. 2019). This would suggest that postexercise cooling may lower postprandial myofibrillar protein synthesis rates. However, this has never been assessed. Therefore, we extend previous work by investigating the impact of postexercise cooling on the incorporation of dietary protein‐derived amino acids within skeletal muscle and subsequent myofibrillar protein synthesis rates. In the present study, we showed that postexercise cooling lowered skin and muscle temperature (compared to the CON leg) immediately after CWI (by ∼20.5 and ∼5°C, respectively) (Fig. 3). We also observed that postexercise cooling lowers the incorporation of dietary protein‐derived amino acids into myofibrillar protein by as much as 26% (Fig. 5) and postprandial myofibrillar protein synthesis rates by almost 20% over the entire 5‐h postexercise recovery period (Fig. 6).

To gain more insight into potential mechanisms that may underlie the observed acute effects of postexercise cooling on postprandial myofibrillar protein synthesis, we assessed the phosphorylation status of several molecular markers that are important in the regulation of myofibrillar protein synthesis (Fig. 8). Apart from a significantly higher phosphorylation of p70S6K^Thr389^ immediately after water immersion in the CWI leg compared to the CON leg, we observed no significant differences between both legs in i.m. signalling proteins that regulate protein translation‐initiation (i.e. mTOR, p70S6K^Thr421/Ser424^, rpS6 and 4E‐BP1). Therefore, these findings with respect to anabolic signalling could not explain the differences we found in myofibrillar protein synthesis between the CWI and CON leg. This could potentially be explained by the fact that our biopsy timing, which was chosen to detect differences in myofibrillar protein synthesis, may not have allowed us to assess transient changes in phosphorylation status of these signalling proteins. Alternatively, it is possible that other factors were responsible for the postexercise CWI induced lowering of postprandial myofibrillar protein synthesis rates, such as a blunting of ribosomal biogenesis (Figueiredo *et al*. 2016). In agreement with the absence in differences in anabolic signalling, we also did not observe differences in mRNA expression of mTOR and p70S6K between the CWI and CON leg (Fig. 9*D* and *E*). Furthermore, when assessing markers of muscle protein breakdown, we did not detect any significant differences between mRNA expression of FOXO1, MuRF1 and MAFbx between the CWI and CON leg (Fig. 9*A–C*). This is in line with a recent study showing that prolonged muscle cooling around resistance exercise does not change the gene expression of these markers of muscle protein breakdown compared to muscle heating (Zak *et al*. 2018). In addition, it was recently shown that there were no clear effects of postexercise CWI on markers of muscle protein breakdown both before and after 7 weeks of resistance‐type exercise training (Fyfe *et al*. 2019). Together, these findings suggest that muscle protein breakdown may not be elevated following postexercise muscle cooling in humans, despite indications in rodents that 24 h of cold‐exposure will increase markers of muscle protein breakdown (Manfredi *et al*. 2013). We also assessed mRNA expression of i.m. transporters involved in glucose (i.e. GLUT4) and amino acid absorption (Fig. 9*F* and 9*I–L*, respectively). We observed no significant differences in GLUT4 mRNA expression between the CWI and CON leg; however, we did observe that some markers (i.e. SNAT2 and CD98) of amino acid transport were significantly lower in the CWI leg compared to the CON leg. This may be either a cause of a lower amino acid uptake in the CWI leg, or simply a consequence of lower blood supply to the cooled muscle tissue, both of which would compromise amino acid uptake, explaining (at least in part) the lower dietary protein‐derived amino acid incorporation in myofibrillar protein in the CWI leg (Fig. 5). Finally, we assessed whether we could detect differences in inflammatory markers (i.e. TNF‐α and IL‐6) between the CWI and CON leg (Fig. 9*G* and *H*) because cooling has been proposed to reduce postexercise inflammation. For mRNA expression of both IL‐6 and TNF‐α, we did not observe any significant reductions in the CWI leg compared to the CON leg, suggesting that postexercise cooling does not reduce (local) i.m. inflammation. In fact, we observed that TNF‐α mRNA expression was significantly higher immediately after water immersion in the CWI leg compared to the CON leg. In line with recent observations by Peake *et al*. (2017), these findings further challenge the notion that postexercise cooling reduces local i.m. inflammation in humans.

In the present study, we show that cooling impairs the capacity of protein feeding to increase myofibrillar protein synthesis rates during acute recovery from exercise. It could be speculated that this merely represents a transient response and that cooling does not have a negative impact on myofibrillar protein synthesis rates when assessed over a more prolonged period. Therefore, in the present study, we applied postexercise cooling over a 2 week period, during which seven consecutive resistance exercise sessions were performed. To assess myofibrillar protein synthesis rates over this entire 2 week period, we applied D_2_O tracer methodology (Fig. 2). This allowed us to measure the incorporation of ^2^H‐labelled alanine into muscle tissue over the entire 2 weeks (Gasier *et al*. 2010). We (Holwerda *et al*. 2018) and others (Wilkinson *et al*. 2014; Brook *et al*. 2015) have previously applied D_2_O tracer methodology to show that resistance‐type exercise training increases daily muscle protein synthesis rates. We now extend on those, as well as on our acute findings, by showing that the repetitive application of postexercise cooling after every exercise session lowers daily myofibrillar protein synthesis rate by ∼12% during a 2 week training period (Fig. 7). The fact that daily myofibrillar protein synthesis is substantially reduced throughout 2 weeks of exercise training would suggest that postexercise cooling negatively affects the skeletal muscle adaptive response to prolonged exercise training. Although some studies have not been able to detect (clear) detrimental effects of postexercise cooling on gains in muscle mass and strength (Ohnishi N, 2004; Yamane *et al*. 2006; Fyfe *et al*. 2019), others have reported attenuated gains in muscle mass and strength as a result of postexercise cooling during prolonged resistance exercise training (Frohlich *et al*. 2014; Roberts *et al*. 2015; Yamane *et al*. 2015).

It could be speculated whether our data would also apply to more elite level athletes. Differences in body composition between recreational and more elite level athletes may modulate the impact of postexercise cooling on muscle temperature (Myrer *et al*. 2001; Stephens *et al*. 2018). In addition, differences in training status may impact postexercise muscle protein synthesis (Damas *et al*. 2015). However, considering that cooling lowered muscle protein synthesis rates by almost 20% during 5 h of postexercise recovery, our findings more than probably translate to more elite level athletes. In support, a long‐term study by Roberts *et al*. (2015) observed attenuated gains in muscle mass and strength when postexercise CWI was applied during 12 weeks of resistance‐type exercise training in well‐trained males.

Our data suggest that postexercise cooling reduces the delivery and/or uptake of dietary protein derived amino acids during acute postexercise recovery. However, more work will be required to confirm this by directly measuring muscle perfusion and/or amino acid uptake in skeletal muscle tissue. Furthermore, the increase in muscle protein synthesis rate during acute postexercise recovery is shown to be severely blunted following cooling. When postexercise cooling is applied consistently during more prolonged exercise training, muscle protein synthesis rates remain lower throughout the entire training period. These data indicate that postexercise cooling attenuates the skeletal muscle adaptive response to exercise training and may therefore compromise exercise‐training efficacy. Individuals aiming to improve skeletal muscle conditioning should therefore reconsider applying cooling as a part of their postexercise recovery strategy.

In conclusion, CWI during recovery from resistance‐type exercise lowers the capacity of the muscle to take up and direct dietary protein‐derived amino acids towards *de novo* myofibrillar protein accretion in healthy, recreationally active males. In addition, when applied consistently CWI during recovery from resistance‐type exercise lowers myofibrillar protein synthesis rates during more prolonged resistance‐type exercise training and, as such, may attenuate skeletal muscle conditioning.

## Additional information

### Competing interests

The authors declare that they have no competing interests.

### Author contributions

This study was conducted at Maastricht University Medical Centre+, Maastricht, The Netherlands. CJF, IWKK, TACV and LJCL conceived and designed the research. CJF and JSJS generated and collected data. CJF and JMS analysed the data. CJF, WDVML, LBV and LJCL interpreted the results. CJF prepared the figures. CJF and LJCL wrote the manuscript. CJF, IWKK, TACV, JSJS, JMS, WDVML, LBV and LJCL revised the manuscript and approved the final version submitted for publication.

### Funding

This study was internally funded by Maastricht University Medical Centre+, Maastricht, The Netherlands.

## Supporting information


**Statistical Summary Document**
