# Supplementary material for: Postexercise cooling impairs muscle protein synthesis rates in recreational athletes
Source: J Physiol. 2019 Dec 29;598(4):755–72. doi: 10.1113/JP278996 (PMC7028023; doi:10.1113/JP278996)
Supplement: Supplementary file 1 — Statistical Summary Document [file TJP-598-755-s001.docx]

**Manuscript Title: Postexercise cooling impairs muscle protein synthesis rates in recreational athletes**

**Authors:** **Cas. J. Fuchs, Imre W.K. Kouw, Tyler A. Churchward-Venne, Joey S.J. Smeets, Joan M. Senden, Wouter D. van Marken Lichtenbelt, Lex B. Verdijk and Luc J.C. van Loon**

**Animal model used, if applicable: N/A**

**Underlying hypothesis:**

1. **We hypothesized that postexercise cooling lowers postprandial muscle protein accretion and subsequent muscle protein synthesis rates during recovery in healthy, young men.**
2. **We hypothesized that postexercise cooling lowers myofibrillar protein synthesis rates during 2 weeks of resistance-type exercise training in healthy, young men.**

**Definitions of ‘n’: For the current research study, *n* = 12 healthy young males.**

**Statistical summary table:**

| Experimental question number* | Finding/ conclusion | Experimental location/ variable  e.g. cortex vs cerebellum or genotype | Mean value  (or other summary statistic) | SD | n (value) | P** | Units | Data comparisons  e.g. WT vs KO | Statistical test | Any other variable  e.g. subjects’ age or sex | Figure/table in which data are presented | Comments  e.g. observation |
| --- | --- | --- | --- | --- | --- | --- | --- | --- | --- | --- | --- | --- |
| 1  Does postexercise cooling (CWI) lower postprandial muscle protein accretion? | Cold-water immersion during recovery from resistance-type exercise does indeed lower postprandial muscle protein accretion. | Muscle from both legs (within subject design) | 0.016 MPE in CWI leg  vs  0.021 MPE in CON leg | CWI: 0.006  vs  CON: 0.007 | n=12 | P=0.016 | MPE = Mole percent excess | CWI (Cold-water immersion)  vs  CON (Thermoneutral water immersion | Students paired T-test. | n/a | Figure 5 | n/a |
| 2  Does postexercise cooling (CWI) lower acute (0-5 hours) postprandial muscle protein synthesis? | Cold-water immersion during recovery from resistance-type exercise does indeed lower muscle protein synthesis rates over 5 hours. | Muscle from both legs (within subject design) | Leucine tracer:  0.058%·h-1 in CWI leg  vs  0.072%·h-1 in CON leg  Phenylalanine tracer:  0.042%·h-1 in CWI leg  vs  0.053%·h-1 in CON leg | Leucine tracer:  0.011%·h-1 in CWI leg  vs  0.017%·h-1 in CON leg  Phenylalanine tracer:  0.009%·h-1 in CWI leg  vs  0.013%·h-1 in CON leg | n=12 | Leucine tracer:  P=0.024  vs  Phenylalanine tracer:  P=0.025 | FSR = fractional synthetic rate (%·h-1 ) | CWI (Cold-water immersion)  vs  CON (Thermoneutral water immersion | Students paired T-test. | n/a | Figure 6 | n/a |
| 3  Does postexercise cooling (CWI) lower muscle protein synthesis rates during 2 weeks of resistance-type exercise training? | Cold-water immersion during recovery from resistance-type exercise does indeed lower muscle protein synthesis rates during 2 weeks of resistance-type exercise training. | Muscle from both legs (within subject design) | 1.48%·d-1 in CWI leg  vs  1.67%·h-1 in CON leg | 0.17%·d-1 in CWI leg  vs  0.36%·d-1 in CON leg | n=12 | P=0.042 | FSR = fractional synthetic rate (%·d-1 ) | CWI (Cold-water immersion)  vs  CON (Thermoneutral water immersion | Students paired T-test. | n/a | Figure 7 | n/a |
